# Identification of Transcriptome-Derived Microsatellite Markers and Their Association with the Growth Performance of the Mud Crab (*Scylla paramamosain*)

**DOI:** 10.1371/journal.pone.0089134

**Published:** 2014-02-13

**Authors:** Hongyu Ma, Wei Jiang, Ping Liu, Nana Feng, Qunqun Ma, Chunyan Ma, Shujuan Li, Yuexing Liu, Zhenguo Qiao, Lingbo Ma

**Affiliations:** 1 East China Sea Fisheries Research Institute, Chinese Academy of Fishery Sciences, Shanghai, China; 2 Key Laboratory of East China Sea and Oceanic Fishery Resources Exploitation, Ministry of Agriculture, Shanghai, China; 3 Yellow Sea Fisheries Research Institute, Chinese Academy of Fishery Sciences, Qingdao, China; Wageningen UR Livestock Research, Netherlands

## Abstract

Microsatellite markers from a transcriptome sequence library were initially isolated, and their genetic variation was characterized in a wild population of the mud crab (*Scylla paramamosain*). We then tested the association between these microsatellite markers and the growth performance of *S. paramamosain*. A total of 129 polymorphic microsatellite markers were identified, with an observed heterozygosity ranging from 0.19 to 1.00 per locus, an expected heterozygosity ranging from 0.23 to 0.96 per locus, and a polymorphism information content (PIC) ranging from 0.21 to 0.95 per locus. Of these microsatellite markers, 30 showed polymorphism in 96 full-sib individuals of a first generation family. Statistical analysis indicated that three microsatellite markers were significantly associated with 12 growth traits of *S. paramamosain*. Of these three markers, locus Scpa36 was significantly associated with eight growth traits, namely, carapace length, abdomen width (AW), body height (BH), fixed finger length of the claw, fixed finger width of the claw, fixed finger height of the claw, meropodite length of pereiopod 2, and meropodite length of pereiopod 3 (MLP3) (*P*<0.05). Locus Scpa75 was significantly associated with five growth traits, namely, internal carapace width, AW, carapace width at spine 8, distance between lateral spine 2 (DLS2), and MLP3 (*P*<0.05). Locus Spm30 was significantly associated with BH, DLS2, and body weight (*P*<0.05). Further analysis suggested a set of genotypes (BC at Scpa36, BC and BD at Scpa75, and AC at Spm30) that have great potential in the selection of *S. paramamosain* for growth traits. These findings will facilitate the development of population conservation genetics and molecular marker-assisted selective breeding of *S. paramamosain* and other closely related species.

## Introduction

The mud crab (*Scylla paramamosain*) is a highly commercially valuable species, mainly distributed along the southeastern coasts of China and other Asian countries such as Japan, Vietnam, and the Philippines. *S. paramamosain* is an important aquaculture and capture marine species in China. Records of *S. paramamosain* aquaculture date back more than 100 years in China [1] and more than 30 years in other Asian countries [2]. Adult *S. paramamosain* mate inshore and the gravid females generally migrate offshore to spawn eggs [3]. *S. paramamosain* has received increasing attention over the years and has been cultured by fishermen because of its wonderful flavor and fast growth rate. The aquaculture production in China reached 110,000 tons in 2011 [4]. However, this production scale does not meet the market demand. An artificial selective breeding program has been launched to develop one or several novel strains with higher economically valuable traits, such as faster growth rates, better flavor, and higher disease resistance.

DNA markers are useful for the assisted breeding of aquacultured organisms. A microsatellite-based parentage assignment technique was successfully developed for *S. paramamosain*, and its success rate for assigning progeny to real parents reaches 95% [5]. Several types of genetic markers, including microsatellites [6]–[9], SNP [10], complete mitochondrial DNA [11], and AFLP [12] have been developed to assist in the improvement and enhancement of the economically important traits of *S. paramamosain*, and the genetic diversity and structure of wild and cultured populations have also been investigated [13], [14]. However, information on the application of molecular technology in the assisted breeding of *S. paramamosain* is limited.

Microsatellites are nuclear genetic markers that are considered an ideal molecular marker system for investigating genetic diversity [15], constructing genetic maps [16], [17], and marker-assisted selection (MAS) [18]. MAS has become a hot topic for aquacultured organisms in recent years, and it can help genetically improve the species by approximately 25% to 50% compared with traditional artificial selective breeding techniques [19]. MAS mainly focuses on selecting target genotypes rather than phenotypes, and it is carried out during the early development of organisms. Few studies have been performed on aquacultured animals. For example, a microsatellite marker is identified to correlate with the disease resistance of a population of giant black tiger shrimp (*Penaeus monodon*) [20]. Four SNPs in giant freshwater prawn (*Macrobrachium rosenbergii*) are significantly associated with three growth traits (body weight, carapace length, and standard length) [21]. A lymphocystis disease-resistant population of Japanese flounder (*Paralichthys olivaceus*) has been developed using microsatellite-assisted selection [18]. Economically important traits associated molecular markers need to be identified to develop better strains of aquatic organisms.

We recently constructed first generation families of *S. paramamosain* and investigated the correlation of different growth traits [22]. In the current study, we initially isolated polymorphic microsatellite markers from a transcriptome sequence library and estimated their genetic variation levels in a wild population. Then, we assessed the association between these markers and the growth performance of this important crab species. This study aims to provide references for population conservation genetics and molecular MAS breeding in *S. paramamosain* and other closely related species.

## Materials and Methods

### Ethics Statement

All animal experiments in this study were conducted according to relevant national and international guidelines. This project was approved by East China Sea Fisheries Research Institute. In China, catching wild mud crab from seawater does not require specific permits. This study does not involve endangered or protected species.

### Sample Collection and Growth Trait Measurement

A wild population of 32 *S. paramamosain* was collected from the coastal waters along Wenchang City, China in September 2011. This population was used to evaluate the polymorphism of transcriptome-derived microsatellite markers. A first generation (G_1_) family of *S. paramamosain* was bred in June 2012 and cultured on Hainan Island, China. The G_1_ crabs were all cultured in the same pond to maintain them under the same environmental condition. A total of 96 full-sib individuals approximately three months of age were randomly collected from the G_1_ family in September 2012. The average weight of these individuals is 82.47 g.

Sixteen growth traits of the 96 full-sib individuals were measured according to Keenan et al. [23] and Gao et al. [24]. These traits included carapace length (CL), carapace width (CW), internal carapace width (ICW), carapace frontal width (CFW), abdomen width (AW), body height (BH), carapace width at spine 8 (CWS8), distance between lateral spine 1 (DLS1), distance between lateral spine 2 (DLS2), fixed finger length of the claw (FFLC), fixed finger width of the claw (FFWC), fixed finger height of the claw (FFHC), meropodite length of pereiopod 1 (MLP1), meropodite length of pereiopod 2 (MLP2), meropodite length of pereiopod 3 (MLP3), and body weight (BW). These 15 morphologic traits were measured to the nearest 0.01 mm using Vernier calipers. Body weight was measured to an accuracy of 0.01 g using a digital electronic balance.

### Genomic DNA Extraction

Genomic DNA was extracted from the muscle tissues of 32 wild and 96 full-sib individuals following the traditional proteinase K and phenol–chloroform extraction method as described by Ma et al. [25]. The concentration of DNA was adjusted to 100 ng/µl, and DNA was stored at −20°C until used.

### Microsatellite Marker Development and Evaluation

In our previous study, we carried out 454 high-throughput pyrosequencing on a mixed cDNA library of *S. paramamosain* from four tissues (muscle, hepatopancreas, eyestalk, and blood) of 12 individuals (the manuscript is being prepared). A total of 540 Mbp of data was produced, and 78,268 unigenes were obtained through sequence similarity with known proteins (E ≤0.00001) in the UniProt and non-redundant (NR) protein databases. A total of 19,011 microsatellites were identified from unigenes using the MISA software under default settings. In the present study, we designed primers for the microsatellite sequences based on the following criteria: a minimum of eight repeats for dinucleotide, trinucleotide, and tetranucleotide repeats and sufficient flanking regions using the software Primer Premier 5.0.

We used a single population of 32 wild individuals to evaluate the polymorphism of the microsatellite markers derived from the transcriptome. Polymerase chain reaction (PCR) was performed using a 12.5 µl total volume that contained 0.4 µM each primer, 0.2 mM each dNTP, 1× PCR buffer, 1.5 mM MgCl_2_, 0.4 units of *Taq* polymerase (TianGen Biotech Co., Ltd, ET101), and approximately 50 ng of DNA. The following conditions were used for the PCR: 1 cycle of denaturation at 94°C for 4 min and 30 cycles of 30 s at 94°C, 50 s at a primer-specific annealing temperature (Table S1), and 50 s at 72°C. In the final step, the products were extended for 7 min at 72°C. The PCR products were separated on 6% denaturing polyacrylamide gel and visualized via silver staining. The allele size was estimated according to the pBR322/*Msp* I marker (TianGen Biotech Co., Ltd, MD206).

### Identification of Growth Traits Associated Microsatellite Markers

All 129 polymorphic microsatellite markers developed from the transcriptome were genotyped in 96 individuals of the G_1_ family, with 30 loci exhibiting polymorphism. We tested the association between the 30 microsatellite loci and 16 growth traits (i.e., CL, CW, ICW, CFW, AW, BH, CWS8, DLS1, DLS2, FFLC, FFWC, FFHC, MLP1, MLP2, MLP3, and BW). The PCRs and electrophoresis were conducted as described above.

### Statistical Analysis

The genetic diversity indices of the microsatellites were calculated using the software POPGENE version 1.31 [26], including the observed number of alleles (*N*_a_), effective number of alleles (*N*_e_), observed heterozygosity (*H*_O_), expected heterozygosity (*H*_E_), polymorphism information content (PIC), Chi-square tests for Hardy–Weinberg equilibrium (HWE), and linkage disequilibrium (LD). Significance values for all multiple tests were corrected through sequential Bonferroni procedure [27].

The associations between microsatellite markers and growth traits (CL, CW, ICW, CFW, AW, BH, CWS8, DLS1, DLS2, FFLC, FFWC, FFHC, MLP1, MLP2, MLP3, and BW) were tested using the General Linear Model (GLM) procedure in the software SPSS version 19. A linear animal model with the fixed effects was used as follows: Y_ijk_ = μ+G_i_+S_j_+e_ijk_, where Y_ijk_ is the observed value of the ijkth trait; μ is the mean value of the trait; G_i_ is the effect of the ith genotype; S_j_ is the effect of the jth sex; and e_ijk_ is the random error effect. All G_1_ individuals used in the association analysis were derived from the same family, cultured under the same conditions, and collected at the same age, so other effects such as batch, generation, family, age, and site were not considered in the statistical model. Significant differences in growth traits among the different genotypes were calculated through multiple comparison analysis using the S-N-K method. Differences with *P* values of 0.05 were considered statistically significant.

## Results

### Characterization of Transcriptome-derived Microsatellite Markers

A total of 563 pairs of primers were successfully designed based on the transcriptome-derived microsatellite sequences. Of these primer pairs, 129 showed polymorphism in a wild population of 32 individuals (Table S1), whereas others exhibited monomorphism, smears, or absence of products. The number of alleles per locus ranged from 2 to 27 (mean = 7), the observed heterozygosity per locus ranged from 0.19 to 1.00 (mean = 0.68), the expected heterozygosity per locus ranged from 0.23 to 0.96 (mean = 0.70), and the PIC per locus ranged from 0.21 to 0.95 (mean = 0.66). Nineteen loci significantly deviated from the HWE after Bonferroni correction (*P*<0.00039), and all loci exhibited no evidence of stuttering and allelic dropout. Furthermore, no significant LD was found in all pairs of loci.

### Genetic Variation in the G_1_ Population

Of the 129 polymorphic microsatellite loci, 30 showed polymorphism in the 96 individuals of the G_1_ family (Table 1), whereas others exhibited monomorphism. A total of 85 alleles were detected, with an average of 2.8 per locus. The observed heterozygosity per locus ranged from 0.25 to 1.00 with an average of 0.74, the expected heterozygosity per locus ranged from 0.22 to 0.75 with an average of 0.56, and the PIC per locus ranged from 0.22 to 0.70 with an average of 0.49. Up to 18 loci demonstrated high polymorphism (PIC >0.5), 9 loci demonstrated intermediate polymorphism (0.25<PIC <0.5), and 3 loci demonstrated low polymorphism (PIC <0.25). No significant LD was detected in all loci pairs.

**Table 1:** Genetic variation of 30 microsatellite markers in G_1_ family of *S. paramamosain.*

Locus	*N*_a_	*N*_e_	*H*_O_	*H*_E_	PIC	Allele frequency			
						A	B	C	D
Scpa19	2	1.3	0.30	0.26	0.22	0.85	0.15	/	/
Scpa32	3	2.7	1.00	0.63	0.55	0.26	0.50	0.24	/
Scpa36	3	2.7	0.75	0.63	0.56	0.25	0.49	0.26	/
Scpa38	3	2.6	0.99	0.62	0.55	0.51	0.28	0.21	/
Scpa40	4	4.0	1.00	0.75	0.70	0.23	0.24	0.27	0.26
Scpa41	2	1.5	0.45	0.35	0.28	0.78	0.22	/	/
Scpa42	3	2.7	1.00	0.63	0.55	0.50	0.26	0.24	/
Scpa43	2	1.3	0.25	0.22	0.20	0.87	0.13	/	/
Scpa46	3	2.7	0.84	0.63	0.55	0.26	0.49	0.26	/
Scpa47	2	1.7	0.57	0.41	0.32	0.72	0.28	/	/
Scpa49	3	2.9	0.90	0.65	0.58	0.32	0.43	0.24	/
Scpa56	2	1.5	0.43	0.34	0.28	0.22	0.78	/	/
Scpa57	2	1.5	0.42	0.33	0.28	0.79	0.21	/	/
Scpa73	3	2.7	0.74	0.63	0.55	0.24	0.26	0.50	/
Scpa74	2	1.4	0.31	0.26	0.22	0.85	0.15	/	/
Scpa75	4	3.8	1.00	0.74	0.69	0.28	0.22	0.18	0.32
Scpa76	3	2.7	0.72	0.64	0.56	0.26	0.48	0.26	/
Scpa77	2	2.0	0.45	0.50	0.37	0.47	0.53	/	/
Spm10	4	3.8	1.00	0.74	0.69	0.21	0.32	0.29	0.18
Spm11	4	3.9	1.00	0.75	0.70	0.22	0.28	0.22	0.28
Spm16	2	1.7	0.55	0.40	0.32	0.73	0.27	/	/
Spm27	2	2.0	0.61	0.50	0.37	0.51	0.49	/	/
Spm30	4	3.9	1.00	0.75	0.70	0.23	0.30	0.20	0.27
Spm35	2	1.8	0.63	0.43	0.34	0.68	0.32	/	/
Spm37	2	2.0	0.67	0.50	0.37	0.53	0.47	/	/
Spm38	4	4.0	1.00	0.75	0.70	0.26	0.23	0.27	0.24
Spm46	4	4.0	1.00	0.75	0.70	0.27	0.25	0.25	0.23
Spm48	3	2.5	0.84	0.60	0.54	0.54	0.23	0.23	/
Spm57	3	2.6	0.75	0.62	0.55	0.28	0.21	0.51	/
Spm61	3	2.9	0.94	0.66	0.58	0.41	0.30	0.29	/
Mean	2.8	2.6	0.74	0.56	0.49	/	/	/	/

*N*_a_, observed number of alleles; *N*_e_, effective number of alleles; *H*_O_, observed heterozygosity; *H*_E_, expected heterozygosity; PIC, polymorphism information content.

### Association between Microsatellite Loci and Growth Traits

Of the 30 polymorphic microsatellite loci in the G_1_ family, Scpa36, Scpa75, and Spm30 were significantly associated with 12 of the 16 growth traits of *S. paramamosain*. Locus Scpa36 was significantly associated with CL, AW, BH, FFLC, FFWC, FFHC, MLP2, and MLP3 (*P*<0.05). At this locus (Table 2), the individuals with genotype BC exhibited the highest phenotypic values for these eight growth traits. Multiple comparisons analysis showed that the individuals with genotype BC grew significantly faster than those with genotype AB in terms of CL, AW, FFLC, FFHC, and MLP2 (*P*<0.05). The individuals with genotype BC showed a significantly faster growth rate than those with genotypes AB and BB in terms of BH (*P*<0.05). The individuals with genotypes BC and AC grew significantly faster than AB individuals in terms of FFWC (*P*<0.05). However, the individuals with genotype BC grew significantly faster than those with AB and BB in terms of MLP3 (*P*<0.05). Meanwhile, the individuals with genotype AC grew significantly faster than those with AB in terms of MLP3 (*P*<0.05).

**Table 2:** Association analysis between microsatellite locus Scpa36 and eight growth traits of *S. paramamosain.*

Locus	Genotype	Number	Growth trait (Means±SD, mm)							
			CL	AW	BH	FFLC	FFWC	FFHC	MLP2	MLP3
Scpa36	AB	19	47.87±7.88^a^	23.85±3.62^a^	28.37±3.96^a^	43.98±7.34^a^	11.80±2.25^a^	17.10±3.37^a^	26.58±3.72^a^	21.88±2.88^a^
	BB	21	49.80±7.85^ab^	24.48±4.08^ab^	28.97±4.68^a^	46.90±9.20^ab^	13.01±3.14^ab^	18.65±4.39^ab^	27.29±4.71^ab^	22.98±4.33^ab^
	AC	23	52.50±8.83^ab^	26.20±4.86^ab^	30.73±5.36^ab^	49.79±9.72^ab^	14.07±3.25^b^	19.99±4.86^ab^	28.70±5.30^ab^	24.73±3.98^bc^
	BC	21	54.46±5.01^b^	27.08±2.75^b^	32.21±3.10^b^	52.37±7.57^b^	14.39±2.68^b^	21.01±4.29^b^	30.45±3.36^b^	25.75±2.64^c^

CL, carapace length; AW, abdomen width; BH, body height; FFLC, fixed finger length of the claw; FFWC, fixed finger width of the claw; FFHC, fixed finger height of the claw; MLP2, meropodite length of pereiopod 2; MLP3, meropodite length of pereiopod 3; ^a, b, ab, bc^ and ^c^, superscripts in different genotypes without a common one are significant different (*P*<0.05).

Locus Scpa75 was significantly associated with traits ICW, AW, CWS8, DLS2, and MLP3 (*P*<0.05). At this locus (Table 3), the individuals with genotype BC exhibited the highest phenotypic values for ICW, CWS8, DLS2, and MLP3. Multiple comparisons analysis showed that the individuals with genotype BC grew significantly faster than those with AD in terms of DLS2 (*P*<0.05). By contrast, no significant differences in phenotypic values were detected between genotype pairs in terms of ICW, AW, CWS8, and MLP3 (*P*>0.05) at this locus.

**Table 3:** Association analysis between microsatellite locus Scpa75 and five growth traits of *S. paramamosain.*

Locus	Genotype	Number	Growth trait (Means±SD, mm)				
			ICW	AW	CWS8	DLS2	MLP3
Scpa75	AD	32	69.79±11.83^a^	24.10±4.20^a^	71.90±12.50^a^	43.45±7.18^a^	22.45±4.20^a^
	AC	17	74.70±11.75^a^	24.98±3.75^a^	77.03±12.06^a^	46.65±6.50^ab^	24.63±3.83^a^
	BD	24	76.82±9.99^a^	26.85±4.32^a^	79.15±10.16^a^	47.21±5.28^ab^	24.54±3.52^a^
	BC	14	77.59±7.34^a^	26.45±2.65^a^	80.00±7.74^a^	48.69±4.30^b^	25.51±2.83^a^

ICW, internal carapace width; AW, abdomen width; CWS8, carapace width at spine 8; DLS2, distance between lateral spine 2; MLP3, meropodite length of pereiopod 3; ^a, b^ and^ ab^, superscripts in different genotypes without a common one are significant different (*P*<0.05).

Locus Spm30 was significantly associated with BH, DLS2, and BW (*P*<0.05). At this locus (Table 4), the individuals with genotype AC exhibited the highest phenotypic values for BH, DLS2, and BW. Multiple comparisons analysis showed that the individuals with genotypes AC and BD grew significantly faster than those with CD in terms of BH and DLS2 (*P*<0.05). The individuals with genotypes AC, BD, and AB grew significantly faster than those with genotype CD in terms of BW (*P*<0.05).

**Table 4:** Association analysis between microsatellite locus Spm30 and three growth traits of *S. paramamosain.*

Locus	Genotype	Number	Growth trait (Means±SD, mm)		
			BH	DLS2	BW
Spm30	CD	18	27.34±4.47^a^	42.13±7.28^a^	57.72±31.66^a^
	AB	24	29.52±5.24^ab^	44.78±7.07^ab^	79.68±38.73^b^
	BD	33	30.88±4.22^b^	47.28±5.30^b^	89.53±32.98^b^
	AC	21	31.08±3.58^b^	47.54±4.25^b^	92.99±31.06^b^

BH, body height; DLS2, distance between lateral spine 2; BW, body weight; ^a, b^ and^ ab^, superscripts in different genotypes without a common one are significant different (*P*<0.05).

No microsatellite marker was significantly associated with CW, CFW, DLS1, and MLP1 (*P*>0.05).

### Microsatellite Markers with the Maximum Potential for Growth Performance Breeding

Of the 16 growth traits, AW was significantly associated with Scpa36 and Scpa75. At locus Scpa36, the average AW (27.08 mm) of the individuals with genotype BC (N = 21) was much higher than that (24.92 mm) of the individuals with the other three genotypes (N = 63). At locus Scpa75, the average AW (26.85 mm) of the individuals with genotype BD (N = 24) was much higher than that (24.86 mm) of the individuals with the other three genotypes (N = 63). Therefore, genotype BC at locus Scpa36 has greater potential in selecting for AW than BD at locus Scpa75.

Trait BH was significantly associated with loci Scpa36 and Spm30. At locus Scpa36, the average BH (32.21 mm) of the individuals with genotype BC (N = 21) was much higher than that (29.43 mm) of the individuals with the other three genotypes (N = 63). At locus Spm30, the average BH (30.96 mm) of the individuals with genotypes AC and BD (N = 54) was much higher than that (28.59 mm) of the individuals with the other two genotypes (N = 42). Therefore, genotype BC at locus Scpa36 would have a more important role in selecting for BH than AC and BD at locus Spm30.

Trait DLS2 was significantly associated with loci Scpa75 and Spm30. At locus Scpa75, the average DLS2 (48.69 mm) of the individuals with genotype BC (N = 14) was much higher than that (45.43 mm) of the individuals with the other three genotypes (N = 73). At locus Spm30, the average DLS2 (47.38 mm) of the individuals with genotypes AC and BD (N = 54) was much higher than that (43.64 mm) of the individuals with the other two genotypes (N = 42). This result shows that genotype BC at locus Scpa75 is better in selecting for DLS2 than AC and BD at locus Spm30.

MLP3 was significantly associated with loci Scpa36 and Scpa75. At locus Scpa36, the average MLP3 (25.75 mm) of the individuals with genotype BC (N = 21) was much higher than that (23.29 mm) of the individuals with the other three genotypes (N = 63). At locus Scpa75, the average MLP3 (25.51 mm) of the individuals with genotype BC (N = 14) was much higher than that (23.64 mm) of the individuals with the other three genotypes (N = 73). Therefore, genotype BC at locus Scpa36 is more useful in selecting for MLP3 than BC at locus Scpa75.

CL, FFLC, FFWC, FFHC, MLP2, ICW, CWS8, and BW were significantly associated with one microsatellite locus. CL, FFLC, FFWC, FFHC, and MLP2 were significantly associated with locus Scpa36. At this locus, the phenotypic values of these five growth traits (54.46 mm, 52.37 mm, 14.39 mm, 21.01 mm, and 30.45 mm, respectively) of the individuals with genotype BC were much higher than those of the individuals with the other three genotypes (50.20 mm, 47.07 mm, 13.03 mm, 18.67 mm, and 27.59 mm, respectively). Therefore, genotype BC at microsatellite locus Scpa36 is useful in selecting for these five growth traits. ICW and CWS8 were significantly associated with locus Scpa75. At this locus, the phenotypic values of the two growth traits of the individuals with genotypes BC and BD (77.10 mm and 79.46 mm, respectively) were much higher than those of the individuals with the other two genotypes (71.49 mm and 73.68 mm, respectively). Therefore, these two genotypes (BC and BD) at locus Scpa75 have great potential in selecting for ICW and CWS8. BW was significantly associated with locus Spm30, and its phenotypic value (92.99 mm) of the individuals with genotype AC was much higher than that (78.74 mm) of the individuals with the other three genotypes. Therefore, genotype AC at locus Spm30 could be useful in selecting for BW in breeding programs.

## Discussion

Next-generation sequencing has recently been used to discover microsatellite markers and SNPs in aquaculture species [28]–[30], and is considered a time-saving, highly efficient approach. Microsatellite markers for the mud crab (*S. paramamosain*) have been reported, but most of them are randomly derived from genomic DNA [6]–[9] and information on known genes is unavailable. This study first reported a large number of transcriptome-level polymorphic microsatellite markers in *S. paramamosain*, which have several advantages compared with DNA-derived microsatellites, such as their correlation with potential known genes, better transferability among different species, and better suitability for comparative mapping and genomic studies [31].

Type I microsatellite markers are predicted to be relatively less polymorphic than those derived from genomic DNA [32], [33]. The genetic diversity indices of the microsatellites isolated in this study (average *N*_a_ of 7.00 and *H*_O_ of 0.68) are nearly equal to those isolated from genes (average *N*_a_ of 5.90 and *H*_O_ of 0.67) [34]. These indices are slightly lower than those discovered from genomic DNA in our previous study (average *N*_a_ of 6.80 and *H*_O_ of 0.76) [9]. A similar phenomenon has been observed in the Pacific oyster (*Crassostrea gigas*) [35]. By contrast, the genetic variation in type I microsatellites is reportedly higher than that of type II markers in the silver crucian carp (*Cyprinus carpio* L.) [36].

Of the 129 microsatellite loci, 30 showed polymorphism in the G_1_ family, whereas the others were monomorphic. A total of 78 effective alleles were detected at 30 loci, which is nearly equal to the observed number of alleles (85). This finding indicates that the alleles in the G_1_ family are uniform distributed, and the variation of microsatellite loci is not significantly affected by selection pressure [37]. In addition, a considerably high genetic diversity was detected in this family (average *H*_O_ of 0.74 and average PIC of 0.49), which is similar to that found in other G_1_ families in a previous study (*H*_O_ ranging from 0.46 to 0.85 and PIC ranging from 0.40 to 0.77) [13], albeit slightly lower than that detected in wild populations (average *H*_O_ of 0.76 and PIC of 0.67) [8].

Scientists have identified a set of microsatellite markers that were significantly associated with growth traits and other economically important traits in aquatic animals [37]–[39]. In the present study, we first analyzed the association between microsatellite markers and the growth traits of *S. paramamosain*, and tried to identify potential markers for molecular MAS. Among the 30 microsatellite markers used in association analysis, Scpa36, Scpa75, and Spm30 were confirmed to be significantly associated with 12 of the 16 growth traits of *S. paramamosain*. All three loci showed high polymorphism in the G_1_ family, with the observed number of alleles ranging from 3 to 4 per locus and the PIC ranging from 0.56 to 0.70 per locus. The loci with low polymorphism (alleles lower than three and PIC lower than 0.5) were not associated with any growth traits (*P*>0.05). This phenomenon indicates that the lowly polymorphic microsatellite loci have less advantage in association analysis than the highly polymorphic loci. A similar trend was found in other aquatic animals such as GIFT (genetically improved farmed tilapia species in China), the common carp (*Cyprinus carpio* L.), and the Japanese scallop (*Patinopecten yessoensis*) [38]–[40].

If a microsatellite marker is closely linked to a phenotypic trait, it would be detected in terms of a significant association according to the theory of linkage disequilibrium [41]. Four QTLs and two microsatellite markers were identified to be significantly associated with growth traits of the half-smooth tongue sole (*Cynoglossus semilaevis*) through the mapping of high-density genetic maps [17]. Eleven significant QTLs related with growth traits and the microsatellite markers associated with these QTLs have been identified in turbot (*Scophthalmus maximus*) [42]. Our study indicates that the microsatellite markers Scpa36, Scpa75, and Spm30 are probably in LD with the QTLs of growth traits of *S. paramamosain*, even though we did not conduct a linkage analysis. The linkage between these markers and QTLs should be confirmed and their genetic distances should be determined through mapping on genetic linkage maps in subsequent studies.

The growth traits AW, BH, DLS2, and MLP3 were significantly associated with two microsatellite loci, and eight traits (CL, FFLC, FFWC, FFHC, MLP2, ICW, CWS8, and BW) were significantly associated with one microsatellite locus. Meanwhile, one microsatellite marker was significantly associated with several different growth traits, and different markers were simultaneously and significantly associated with one trait. This phenomenon indicates that one locus contributes to multiple growth traits and multiple loci influenced the same growth trait of *S. paramamosain*. Similar cases have also been found in other animals, such as the largemouth bass (*Micropterus salmoides*) [43] and the swimming crab (*P. trituberculatus*) [37]. As far as we know, growth traits are quantitative traits and they are possibly controlled by several to numerous genes. These genes may have segregated and/or recombined among different generations. Hence, we should investigate the replicability of these three markers in different families and populations, and evaluate their correlation across different generations. In artificial breeding programs, individuals with the target genotypes of these three microsatellite loci should be chosen as candidate parents for breeding, and their offspring that carry the target genotypes should be chosen again. These target microsatellite loci will thus be applied for the practical selection of *S. paramamosain* for growth performance.

## Conclusions

We initially isolated 129 transcriptome-derived polymorphic microsatellite markers. We then identified three markers that were significantly associated with 12 phenotypic growth traits of the mud crab (*Scylla paramamosain*). These findings are helpful in investigations on the population conservation genetics, construction of genetic maps, and molecular MAS of *S. paramamosain* and other closely related species.

## Supporting Information

Table S1 Characterization of 129 polymorphic microsatellite markers derived from a transcriptome sequence library in *S. paramamosain.*
(DOCX)
