# Supplementary material for: Identification of Transcriptome-Derived Microsatellite Markers and Their Association with the Growth Performance of the Mud Crab (Scylla paramamosain)
Source: PLoS One. 2014 Feb 13;9(2):e89134. doi: 10.1371/journal.pone.0089134 (PMC3923883; doi:10.1371/journal.pone.0089134)
Supplement: Table S1 — Characterization of 129 polymorphic microsatellite markers derived from a transcriptome sequence library in S. paramamosain. (DOCX) [file pone.0089134.s001.docx]

**Table S1** Characterization of 129 polymorphic microsatellite markers derived from a transcriptome sequence library in *S. paramamosain.*

| Locus | Repeat sequence | Primer sequence ( 5’ - 3’) | *T*_a_ (℃) | *N*_a_ /*N*_e_ | Allele size range (bp) | *H*_O_ | *H*_E_ | PIC | *P* | GenBank accession no. |
| --- | --- | --- | --- | --- | --- | --- | --- | --- | --- | --- |
| Scpa13 | (AC)_21_T(CA)_8_ | GCGAGGCGATAACTGGAT  TAAGTACCATTTATTTGATAACCCA | 51 | 8/5.3 | 242-316 | 0.77 | 0.83 | 0.79 | 0.027 | KC793905 |
| Scpa14 | (TG)_19_ | CTATTGCTCCTCCTTTGTATC  TTACTAATAAGTTATGCTGACTCAT | 51 | 9/6.0 | 199-273 | 0.58 | 0.85 | 0.81 | 0.040 | KC793906 |
| Scpa15 | (TG)_14_ | CCCCACCCGTCTATGCTA  CACTACTTCGGTGTCCACTTTTA | 55 | 9/7.1 | 290-312 | 0.81 | 0.87 | 0.84 | 0.673 | KC793907 |
| Scpa16 | (GA)_10_ | CATCAGAGCACATCCCCAG  GGCTCGCATCTTGTCGTG | 54 | 9/5.9 | 312-358 | 0.90 | 0.84 | 0.81 | 0.848 | KC793908 |
| Scpa17 | (CT)_8_ | AGCAGTCCACCATACAGG  GTCAGAGAAAGATAAAAGAGAAC | 53 | 3/1.3 | 265-277 | 0.19 | 0.23 | 0.21 | 0.021 | KC793909 |
| Scpa18 | (AT)_11_ | AGTCTACAAGGGTCGCAAGT  GAATGAACGAAGCAAGCC | 49 | 9/7.2 | 260-308 | 0.94 | 0.88 | 0.85 | 0.605 | KC793910 |
| Scpa19 | (AC)_9_ | ACAAACATTGCCACTCAT  ACCTGAAAAGAGGAAACTAA | 45 | 5/3.6 | 236-248 | 0.68 | 0.73 | 0.68 | 0.018 | KC793911 |
| Scpa20 | (AC)_12_ | TCTCTTCAAAGCAACACAGATGGT  ACAGGGTGGGGGTGGGTA | 56 | 7/4.6 | 178-202 | 0.97 | 0.80 | 0.75 | 0.144 | KC793912 |
| Scpa21 | (AAT)_18_(AAC)_6_ | AAACAAAAACTTTACTGGATACAT  GCTTGTCATCCTAAGTGGG | 50 | 8/6.5 | 276-303 | 0.84 | 0.86 | 0.83 | 0.270 | KC793913 |
| Scpa22 | (TAC)_10_ | ACACCTGCTACTGAACAACA  AACAAGAAGGAGGATAAGAAC | 51 | 5/2.8 | 299-314 | 0.56 | 0.65 | 0.58 | 0.000^*^ | KC793914 |
| Scpa23 | (TAG)_10_ | AAGCACCAAGAGGAGAGT  CTCATGTCTCGCTACACC | 53 | 10/5.8 | 222-276 | 0.87 | 0.84 | 0.81 | 0.703 | KC793915 |
| Scpa24 | (AGT)_12_ | TAGGGCTGAAATGTATGAAA  AATGAGGCTAAGAATGTGAAT | 46 | 11/6.1 | 275-317 | 0.88 | 0.85 | 0.82 | 0.565 | KC793916 |
| Scpa25 | (GTAT)_10_ | GAGGCATTGGTGAGCAGTT  CCATTGTCGTATCCCTTTGA | 49 | 11/3.8 | 250-310 | 0.69 | 0.75 | 0.71 | 0.065 | KC793917 |
| Scpa26 | (TAG)_14_ | CCAAGGAAGGAACAGGAAA  CCAGAAACACACTGAATGCT | 50 | 13/10.0 | 275-320 | 0.94 | 0.91 | 0.89 | 0.000^*^ | KC793918 |
| Scpa27 | (AG)_12_ | GGGCACGTTAAGATACAA  AAGGCTGACTGGAAACTC | 52 | 14/8.7 | 265-307 | 0.91 | 0.90 | 0.88 | 0.818 | KC793919 |
| Scpa28 | (AC)_8_ | TAGGGTGTATTATTATTTAGGTTA  CGAAGTTTATTTTGTCCTGT | 48 | 3/2.4 | 231-243 | 0.50 | 0.59 | 0.51 | 0.200 | KC793920 |
| Scpa29 | (GT)_14_ | GTGAAACAAAAGTGGCAATGA  TGAACACAGCCCTTGATGG | 48 | 5/4.0 | 200-216 | 0.91 | 0.76 | 0.71 | 0.018 | KC793921 |
| Scpa30 | (TG)_9_ | GGAGGTTTGTCTTTTGAA  GTCATCTTTGCTCTGTTCTA | 47 | 6/2.2 | 237-265 | 0.69 | 0.55 | 0.52 | 0.910 | KC793922 |
| Scpa31 | (TC)_11_ | CCAGGTAAGGTAACACATTGAACA  TTGTTTCTGCTGCGGCTC | 50 | 3/2.0 | 320-346 | 0.58 | 0.50 | 0.43 | 0.242 | KC793923 |
| Scpa32 | (CA)_13_ | TCTGAGGCTTGCCTAATC  TCTGTCTGGAAACAACTGG | 50 | 14/11.5 | 192-234 | 0.84 | 0.93 | 0.91 | 0.288 | KC793924 |
| Scpa33 | (CA)_13_ | GTTCCTGCGTGGGTGCTC  ACATCTAATCCAACCATGCGTAC | 56 | 11/6.5 | 205-241 | 0.69 | 0.86 | 0.83 | 0.033 | KC793925 |
| Scpa34 | (GT)_27_ | CCTCGTTTCAGATTTGGTTGT  CGACTAATCCCCCCACAAT | 50 | 13/9.6 | 283-309 | 0.94 | 0.91 | 0.89 | 0.264 | KC793926 |
| Scpa35 | (TG)_13_ | TGGTGGTGATGTTCGTGT  ATCTGTTGTAGTAAGGCTGC | 50 | 4/1.5 | 240-248 | 0.19 | 0.33 | 0.30 | 0.004 | KC793927 |
| Scpa36 | (GA)_8_A(AG)_9_ | GTTAGTCCGTTGTATTATCTTTGTC  GGTTAGTGCTTGGATGGGT | 55 | 10/2.1 | 164-210 | 0.44 | 0.54 | 0.52 | 0.000^*^ | KC793928 |
| Scpa37 | (TG)_17_ | AGATGAGATCGCCAAGGGT  CTGACCTGATTGTGTAACCCA | 53 | 7/4.5 | 242-276 | 0.58 | 0.79 | 0.75 | 0.000^*^ | KC793929 |
| Scpa38 | (AC)_10_ | GGCTACCTGTATCTAAATGAA  CGTGAATAGATAGAGTTATGATG | 48 | 6/2.9 | 164-178 | 0.69 | 0.67 | 0.60 | 0.835 | KC793930 |
| Scpa39 | (TC)_11_ | GTCTGTCCAGGAGCACTAAC  AATGGTAATATGCTTATGTTTTT | 43 | 4/3.0 | 222-230 | 0.52 | 0.68 | 0.60 | 0.015 | KC793931 |
| Scpa40 | (ATG)_8_(ATA)_10_ | TTGTTGTGAGGGATTAGTATATTG  GTGACTGTCGGCTGGGTA | 50 | 9/4.2 | 238-274 | 0.88 | 0.77 | 0.73 | 0.757 | KC793932 |
| Scpa41 | (CAG)_7_ | TCCATAGAGCCCTTACAG  AGTATTTGTTGGCAGAAGA | 48 | 8/2.8 | 160-184 | 0.69 | 0.65 | 0.57 | 0.999 | KC793933 |
| Scpa42 | (TA)_8_ | ATTGTAAATGTAAACCTCGTAACT  GAATACTGCTCTATGGAACTACTAA | 50 | 7/5.0 | 240-272 | 1.00 | 0.81 | 0.77 | 0.289 | KC793934 |
| Scpa43 | (GT)_10_ | CAGTTTGGGGGTTTGAGT  CATCTGAGAGCCCTTTGTTA | 47 | 12/8.0 | 244-290 | 0.90 | 0.89 | 0.86 | 0.647 | KC793935 |
| Scpa44 | (TTG)_9_ | GTCTTCTGTTGCGCTGCTA  CTTGACGCTGCTCATTCG | 52 | 11/6.1 | 248-311 | 0.69 | 0.85 | 0.82 | 0.033 | KC793936 |
| Scpa45 | (TG)_12_ | CAGAGAAATGAAAAAAAATCG  CATTGATGAGGAGGGGAC | 45 | 2/1.9 | 192-200 | 0.72 | 0.47 | 0.35 | 0.002 | KC793937 |
| Scpa46 | (GT)_10_ | CTGGAGCAGAATGGAATAG  TGTACACACTCATCCTGTAAGAT | 52 | 5/3.1 | 185-195 | 0.71 | 0.69 | 0.62 | 0.968 | KC793938 |
| Scpa47 | (GAGT)_10_ | GGTGGAAGTGAAGTCTGG  TGTTGCTGCCTACGATTA | 50 | 6/4.2 | 299-323 | 0.81 | 0.78 | 0.73 | 0.145 | KC793939 |
| Scpa48 | (AGGAC)_8_(AGGGT)_12_ | AGTGTAAGGTGGGATTATGTC  CTTGAATTGCCTTGCTTC | 47 | 27/19.3 | 146-291 | 0.78 | 0.96 | 0.95 | 0.008 | KC793940 |
| Scpa49 | (CA)_10_ | TATTTTCTTACGGCACGAAGTTAC  TCCTGGGCTGACGGATTT | 55 | 9/2.7 | 272-344 | 0.72 | 0.64 | 0.56 | 0.999 | KC793941 |
| Scpa50 | (GAT)_12_ | GAAACTCAAGCAAAGACAA  CTCTATTTTTTTGATAATTTCTC | 43 | 11/7.2 | 206-239 | 0.87 | 0.88 | 0.85 | 0.889 | KC793942 |
| Scpa51 | (GT)_8_A(TG)_6_TAC(GT)_7_ | AAGTACCACCAATAAATATGAATAA  TGGAAAAAATAACATAAATAAAAGT | 46 | 8/5.6 | 262-280 | 0.63 | 0.83 | 0.80 | 0.102 | KC793943 |
| Scpa52 | (TG)_9_ | GTGCACATGACATGATCTA  CGTGGTGTTGTTGTTAAT | 49 | 14/9.3 | 160-206 | 0.83 | 0.90 | 0.88 | 0.092 | KC793944 |
| Scpa53 | (GT)_8_ | GCTTCTGCCGCCACAATG  CCCTAACTGCCCTACAGCTCTG | 59 | 6/4.7 | 290-320 | 0.97 | 0.80 | 0.76 | 0.258 | KC793945 |
| Scpa54 | (CCA)_8_ | TTTATTCATTATTTCTTTTCTGTAT  AGAAGACGAGGGGTAAGG | 43 | 4/3.2 | 274-289 | 0.59 | 0.70 | 0.63 | 0.609 | KC793946 |
| Scpa55 | (AG)_8_ | GCCTGTACTACTTATTCCATTCTT  CTCGCAAACACCACCAAC | 54 | 4/1.3 | 162-170 | 0.25 | 0.23 | 0.22 | 0.997 | KC793947 |
| Scpa56 | (CAC)_8_ | ACGAAGAAATAAAGGAAGATGAG  GGAACCGAAACCTGAACC | 54 | 7/3.6 | 242-263 | 0.97 | 0.73 | 0.68 | 0.197 | KC793948 |
| Scpa57 | (AC)_9_ | GAGTGTCTGGGAATGGAAT  TCACGGTATGAAGGTTGG | 54 | 5/2.5 | 233-243 | 0.69 | 0.62 | 0.56 | 0.904 | KC793949 |
| Scpa58 | (TG)_16_ | AATGTTGTGACTTACCTTTGA  TTCCTACACTATCATAGCCAG | 46 | 8/5.0 | 273-307 | 0.80 | 0.81 | 0.77 | 0.172 | KC793950 |
| Scpa59 | (CA)_12_ | CCTCATACTTTTTTTTATTTGG  GTGGTATCAACGCAGAGTG | 43 | 5/4.3 | 236-246 | 0.63 | 0.78 | 0.73 | 0.305 | KC793951 |
| Scpa60 | (AT)_10_ | ACGGCTGAGCACGGCAAT  TTTCTGTTCTGATGTCTGGTGGTTT | 57 | 5/4.0 | 392-320 | 0.37 | 0.76 | 0.71 | 0.000^*^ | KC793952 |
| Scpa61 | (TG)_8_ | GTAGTTACTTGAATAAAGGGAAT  CTTGAGCCTCTTGTGGAA | 46 | 5/3.1 | 180-194 | 0.42 | 0.69 | 0.62 | 0.018 | KC793953 |
| Scpa62 | (AC)_9_ | TATTTGTTCCAAAACCAGTT  AAGCCCTTCCAATGTATG | 46 | 5/1.9 | 217-241 | 0.34 | 0.47 | 0.42 | 0.000^*^ | KC793954 |
| Scpa63 | (TG)_9_ | AAACAATAATAAAAGACAAGCA  ATTTAGGTAGACAATAACAGTAGC | 46 | 9/6.2 | 238-256 | 0.42 | 0.85 | 0.82 | 0.000^*^ | KC793955 |
| Scpa64 | (CA)_9_ | CCAGTCTAAAAGCACCAAC  CCAATCAAACTCCCAAAT | 43 | 5/2.5 | 275-293 | 0.31 | 0.61 | 0.54 | 0.008 | KC793956 |
| Scpa65 | (AC)_8_ | GGTAGTGTGAGTGTACGATGAA  CTGTATGTCTCTATGTAGGTGTTGT | 58 | 14/10.0 | 170-218 | 0.97 | 0.92 | 0.89 | 0.007 | KC793957 |
| Scpa66 | (TG)_8_ | AAGTGGTATCAACGCAGAG  CAGGCAGCAGGGGAAGGT | 51 | 5/3.3 | 257-271 | 0.50 | 0.71 | 0.64 | 0.000^*^ | KC793958 |
| Scpa67 | (AT)_9_ | CCTGTTTTATGCAGAGCC  GACGCCTTACATTTACGC | 52 | 5/3.6 | 289-299 | 0.68 | 0.73 | 0.68 | 0.077 | KC793959 |
| Scpa68 | (TG)_10_ | AACAAGGAAAAAGGAAGAACAT  AGCGCACCACAGGATAAA | 52 | 10/6.6 | 171-193 | 0.84 | 0.86 | 0.83 | 0.153 | KC793960 |
| Scpa69 | (ACC)_8_ | CAAATGTAGAGGGACGAAATAGA  CTGCTGCTGTCGCTGATG | 47 | 9/6.4 | 320-371 | 1.00 | 0.86 | 0.83 | 0.195 | KC793961 |
| Scpa70 | (TAG)_9_ | TGTAGAAGAAAACGGTGGAC  AGGAACATAATAGCAATAACGA | 50 | 8/2.4 | 211-241 | 0.52 | 0.60 | 0.56 | 0.036 | KC793962 |
| Scpa71 | (TAA)_9_ | AAGATTTCCACGTTTGATT  CTCATACCACCATCTTCACT | 47 | 7/2.1 | 217-238 | 0.34 | 0.53 | 0.50 | 0.000^*^ | KC793963 |
| Scpa72 | (ATT)_14_ | TGCCGTCATTACTTCGTC  GAGAGTGCTTTGCATTATCA | 52 | 15/10.7 | 239-287 | 1.00 | 0.92 | 0.90 | 0.118 | KC793964 |
| Scpa73 | (TC)_8_ | TATTCACGGGTGTCTTCG  GATTATCCCACGCTTTCA | 52 | 13/7.1 | 214-290 | 0.81 | 0.87 | 0.85 | 0.000^*^ | KC793965 |
| Scpa74 | (TG)_8_ | CTTGATTTCTAGTTAGTTTACCG  GTTCCTTGTATGTAAAGTGTCC | 49 | 6/1.9 | 187-221 | 0.50 | 0.49 | 0.43 | 0.957 | KC793966 |
| Scpa75 | (ATA)_8_ | CGAAAATAGAGTAAAACC  GTTTTTATGTTGTAATTGAC | 51 | 12/3.8 | 160-205 | 0.78 | 0.75 | 0.70 | 0.987 | KC793967 |
| Scpa76 | (TAG)_11_ | TATTATCCTGTCATCCTA  GACTCCAACTTTGTATTA | 43 | 15/8.8 | 172-226 | 0.90 | 0.90 | 0.88 | 0.943 | KC793968 |
| Scpa77 | (CA)_13_ | GTCTTTCAGGGTAGAGGTTATTA  CACAACAGATGAAGAACGCT | 54 | 12/7.5 | 275-361 | 0.81 | 0.88 | 0.85 | 0.017 | KC793969 |
| Scpa78 | (CAG)_5_ | GTAGCTCAAGGTTAAGGAAGGAT  TTATGCCACTTACTTGACTTATGC | 53 | 2/1.6 | 228-237 | 0.53 | 0.40 | 0.31 | 0.048 | KC793970 |
| Scpa79 | (GAG)_9_ | ATGAGCATCACAGGCAAG  TAAGTCACCCAAGTTATTTTC | 49 | 9/6.1 | 207-261 | 0.88 | 0.85 | 0.82 | 0.004 | KC793971 |
| Spm01 | (TAT)_15_ | AGCAGGACAAGGCGAGTG CTTTGCTTGACGCTTTCG | 56 | 5/4.1 | 349-364 | 0.56 | 0.77 | 0.71 | 0.228 | JX102573 |
| Spm02 | (AC)_8_ | AGCAGCCTTTCAGGTATG AACGCAGAGTGACCATTAC | 56 | 6/2.6 | 300-312 | 0.56 | 0.63 | 0.59 | 0.468 | JX102574 |
| Spm03 | (AC)_9_ | ATAAACGTGCGGCACTGC GCGTCTGTAAAGGGTAATTC | 56 | 4/3.3 | 113-121 | 0.47 | 0.71 | 0.64 | 0.008 | JX102575 |
| Spm04 | (CTGT)_6_ | AATTTGGCTGTTTATTTCTG  GAGGTACTTCGATTTTGA | 50 | 8/6.0 | 76-108 | 0.74 | 0.85 | 0.81 | 0.494 | JX102576 |
| Spm05 | (TGG)_7_ | AGTAACTGCTGATGAGGGAG TCGGCACAAATACTCTACAA | 50 | 3/1.4 | 326-335 | 0.31 | 0.28 | 0.25 | 0.807 | JX102577 |
| Spm06 | (TAA)_9_ | ACTCTGCCTTCTCCGTTAT AAAGGAAGAGCCCTGGTA | 50 | 5/3.9 | 239-254 | 0.53 | 0.75 | 0.70 | 0.014 | JX102578 |
| Spm07 | (TG)_8_TT(TG)_7_ | GATAGATAACCCTGACCTTTAGTGA TTTGCCTTTTTGTTTTACCTGT | 58 | 4/2.8 | 90-98 | 0.78 | 0.66 | 0.59 | 0.008 | JX102579 |
| Spm08 | (TA)_7_ | ATGGGCAAATCAAATGCGAAT ACGGCTGAGCACGGCAAT | 58 | 7/3.3 | 207-223 | 0.56 | 0.71 | 0.65 | 0.581 | JX102580 |
| Spm10 | (AC)_6_ | TGAGATACATTTGTTCCTGA CTTTCTTAAAACCGCGAG | 56 | 6/3.9 | 179-195 | 0.68 | 0.75 | 0.71 | 0.231 | JX102581 |
| Spm11 | (TA)_7_ | CGGGGCAGTAGCAAGTTTAA TCCTTTGATTTCTGGGTGTCTC | 60 | 8/6.8 | 129-149 | 0.77 | 0.87 | 0.84 | 0.153 | JX102582 |
| Spm12 | (CAC)_13_ | GGAGGGTGGGGCTGCTTG TACAGAAGACGCAAGTGACAAGGTT | 60 | 4/3.0 | 255-270 | 0.88 | 0.68 | 0.60 | 0.087 | JX102583 |
| Spm13 | (CAC)_8_ | GGCGGATGAGGAAATGGA TTAGTGATGATGGTTGCAGGG | 59 | 4/2.2 | 196-211 | 0.56 | 0.55 | 0.44 | 0.808 | JX102584 |
| Spm14 | (TC)_8_ | AATAAGACACAGACATAAGCATAAA CCCGTGTCAGCCATGGT | 50 | 3/2.1 | 204-212 | 1.00 | 0.52 | 0.40 | 0.000 | JX102585 |
| Spm15 | (CT)_7_ | CTCCTCATTCCCTAGACACG TTGGTTCTTTGGCATCCT | 50 | 3/2.6 | 232-238 | 1.00 | 0.63 | 0.55 | 0.000^*^ | JX102586 |
| Spm16 | (AC)_8_ | GCGTAAGTCATTATTCAGTTC TTGTCCTCATTCCTCGTC | 50 | 5/2.0 | 205-217 | 0.47 | 0.50 | 0.46 | 0.000^*^ | JX102587 |
| Spm17 | (GA)_8_ | AGGGCGTGTTCATTATTC TTTATCAGTCTATCGCTCTAT | 48 | 8/5.5 | 123-157 | 0.97 | 0.83 | 0.80 | 0.016 | JX102588 |
| Spm18 | (AG)_8_ | GATGCTTGAGCCAAAAATA GTAGATGGGTTACAATGTGACT | 48 | 8/4.4 | 128-160 | 0.79 | 0.79 | 0.74 | 0.837 | JX102589 |
| Spm19 | (GT)_8_ | TTACTCGGATGATTTCGG CCAGCCAGGCATTATTCT | 48 | 13/9.8 | 290-320 | 0.81 | 0.91 | 0.89 | 0.107 | JX102590 |
| Spm20 | (GAT)_10_ | AAGTAGTATTCTGGTGCTGAC CATCATGGGTTTATCTTACA | 53 | 13/8.3 | 216-273 | 0.91 | 0.89 | 0.87 | 0.072 | JX102591 |
| Spm21 | (CTGT)_7_CTGT(CTGC)_5_ | TGCTGGCATACTGGAGGT GACTACACAGATCATTAATTGGC | 53 | 7/3.5 | 221-269 | 0.81 | 0.72 | 0.68 | 0.393 | JX102592 |
| Spm22 | (GGT)_7_ | CATCTAATCCAACCATGCGTAC CAGGCTGTTCCTTCGTTCC | 55 | 5/4.2 | 258-276 | 0.55 | 0.77 | 0.72 | 0.008 | JX102593 |
| Spm23 | (TA)_6_ | GCAGTATTTATGAGTGTAGGGAGA CCAATTAGCCTCGTTTGC | 54 | 4/2.6 | 202-210 | 0.70 | 0.62 | 0.55 | 0.632 | JX102594 |
| Spm24 | (TAA)_9_ | ATTTCCACGTTTGATTAGAAAGAAG CACCATCTTCACTATCATCTCGC | 54 | 3/1.8 | 201-210 | 0.38 | 0.46 | 0.41 | 0.222 | JX102595 |
| Spm25 | (TAAT)_11_ | AAGTGACATAGCAGCAATACC CAGAGGCCATGTTTGTTTAT | 53 | 6/3.0 | 290-318 | 0.58 | 0.68 | 0.62 | 0.421 | JX102596 |
| Spm26 | (CA)_19_ | CTAGCGTCCTCGGTCTTG TACCTACATGGTGCCATCTTA | 53 | 6/3.5 | 180-196 | 0.61 | 0.72 | 0.66 | 0.023 | JX102597 |
| Spm27 | (TTA)_7_TC(ATT)_7_ | ACAGCGAGTGCTTTCTTTCA TCTGCCTAGCCAGGGATC | 56 | 3/1.5 | 260-275 | 0.35 | 0.34 | 0.29 | 0.966 | JX102598 |
| Spm28 | (TAAT)_6_ | GGGCTAGACCCTACCAAGT TGGGTGGAAATAACTGCATAA | 51 | 5/3.0 | 262-286 | 0.52 | 0.68 | 0.63 | 0.052 | JX102599 |
| Spm29 | (CA)_6_ | CAGTGGCGAAGGGATGAA TTAGGTAGTGTTGTGAATGGGAT | 51 | 3/2.0 | 305-313 | 0.50 | 0.51 | 0.45 | 0.037 | JX102600 |
| Spm30 | (AG)_6_ | GGAGAAGTGGGACGAACA AGACGAATGGCACAACAA | 51 | 3/3.0 | 261-267 | 0.44 | 0.68 | 0.59 | 0.020 | JX102601 |
| Spm31 | (GAG)_8_ | AACGCCCTCCCACATAAC TCTGCCAGCTTCTTTCCA | 52 | 3/2.5 | 246-258 | 1.00 | 0.60 | 0.51 | 0.000^*^ | JX102602 |
| Spm32 | (ATG)_6_ | TAACGCAAGCCAGGTGTC CCAATACAATTAAGTCCGACAA | 52 | 4/2.2 | 202-217 | 0.44 | 0.55 | 0.49 | 0.036 | JX102603 |
| Spm33 | (AC)_15_N_47_(TC)_6_(AC)_12_ | TCAGGCACCCAGAACACC ATTCGCTACCCATAACTCCC | 52 | 3/2.5 | 283-309 | 0.96 | 0.61 | 0.52 | 0.000^*^ | JX102604 |
| Spm34 | (AG)_8_ | ACAACTTTCTGACTCACCCTG TCCTCCCTTTCTTTCCCT | 52 | 3/2.9 | 150-178 | 0.78 | 0.66 | 0.58 | 0.182 | JX102605 |
| Spm35 | (TCA)_7_ | TTGTGGTACTGACTTTATTTGTT  TGTGTGTTAAGTTACCAGTGTTT | 52 | 5/1.8 | 160-175 | 0.47 | 0.45 | 0.42 | 0.312 | JX102606 |
| Spm36 | (AC)_13_ | CAGTGGTATCAACGCAGAG AGGATTAAACAAGAGGGACA | 52 | 5/2.4 | 160-172 | 0.50 | 0.59 | 0.49 | 0.955 | JX102607 |
| Spm37 | (TGGTG)_5_ | AGCATCCCAGCCAGTCAT TTCAAATCATACCACCCTTA | 50 | 2/2.0 | 221-236 | 0.65 | 0.50 | 0.37 | 0.111 | JX102608 |
| Spm38 | (AC)_20_ | TGGCACTCAGTAGAAGAT CGTGTACGTAAGAATTAATAG | 50 | 9/4.3 | 223-247 | 0.66 | 0.78 | 0.75 | 0.256 | JX102609 |
| Spm39 | (GTG)_10_ | TATATACTGCTCTGCTTCAT TTTATTGGTGGTACTTGC | 50 | 4/1.8 | 135-150 | 0.33 | 0.46 | 0.40 | 0.002 | JX102610 |
| Spm40 | (AC)_10_ | CTTTCCACGCTTCATAAT ATGTAAGGAAGATTTAAGAGC | 46 | 6/3.6 | 190-204 | 0.66 | 0.74 | 0.69 | 0.111 | JX102611 |
| Spm41 | (TGT)_8_N_52_(GTT)_5_ | TTTTATTGTGGCGGAATC TTTGGTGGAAAGTGAAGGT | 48 | 6/5.4 | 165-287 | 0.46 | 0.83 | 0.79 | 0.000^*^ | JX102612 |
| Spm42 | (TAA)_9_ | GAAGTATTTCCACGTTTGA CATCTTCACTATCATTACGC | 50 | 4/1.8 | 210-225 | 0.40 | 0.45 | 0.40 | 0.653 | JX102613 |
| Spm43 | (GTATTG)_6_ | TTCTGGCTCCTCAACCTC CTATCACCAACGCCCTTT | 56 | 4/2.5 | 162-192 | 0.84 | 0.61 | 0.53 | 0.028 | JX102614 |
| Spm44 | (AC)_10_N_8_(TC)_6_ | ATGGTGATATATGAAGCAGAGC TGAAAGAGGGAGAGACAGAGA | 56 | 7/4.7 | 260-278 | 0.63 | 0.80 | 0.76 | 0.065 | JX102615 |
| Spm45 | (GT)_11_ | GTCTCAGGATTAGAGGAGGTGC GTTGTTAGCTGGAATCTACCTGT | 56 | 5/4.1 | 231-247 | 0.77 | 0.77 | 0.72 | 0.099 | JX102616 |
| Spm46 | (AC)_16_ | CCTAATCTAACTTTACCTTGCT TGTAACTCATCACTTCCAGAC | 51 | 8/6.2 | 243-265 | 0.94 | 0.85 | 0.82 | 0.050 | JX102617 |
| Spm47 | (GA)_7_N_32_(AG)_7_ | TGCTTGCCTGAGTTTACC ACACTCACAAACCCCAAC | 51 | 4/4.0 | 110-120 | 0.96 | 0.76 | 0.70 | 0.000^*^ | JX102618 |
| Spm48 | (AT)_8_ | GTGGGGAGCAAGCAACGA TCAATCATACCTGAATTACAAAAGG | 52 | 6/3.7 | 251-267 | 0.59 | 0.74 | 0.70 | 0.004 | JX102619 |
| Spm49 | (GTT)_9_ | GGATGACAGAATGGGTGAAA ACTGCTGATGATATAGGAAAACA | 52 | 4/1.5 | 237-252 | 0.41 | 0.35 | 0.33 | 0.929 | JX102620 |
| Spm50 | (CA)_10_N_23_(AATA)_8_ | GCAAATCTGCTCCGTCTC ATTAGCAGCATTAGTAGTAGATTTC | 52 | 5/1.7 | 282-318 | 0.29 | 0.43 | 0.40 | 0.172 | JX102621 |
| Spm51 | (AC)_17_ | CAGTGGTATCAACGCAGAG AGGATTAAACAAGAGGGACA | 52 | 5/3.2 | 160-174 | 0.88 | 0.70 | 0.63 | 0.085 | JX102622 |
| Spm52 | (CT)_9_ | ATCTTATGCTGCTGCTCTT AGGCGTGACTTGTCTGTG | 55 | 10/8.9 | 280-320 | 0.84 | 0.90 | 0.88 | 0.360 | JX102623 |
| Spm53 | (AC)_16_ | GTGACATACAGTAATCCAAACCT TGAGGAACACTGAGCAACAT | 51 | 11/7.1 | 201-265 | 0.93 | 0.88 | 0.85 | 0.285 | JX102624 |
| Spm54 | (AG)_11_ACAGAC(AG)_5_ | GAATGAACTCACAATGAAGGAT GGGTACCAGGTAAGGTAACAC | 49 | 3/1.8 | 160-170 | 0.55 | 0.47 | 0.41 | 0.585 | JX102625 |
| Spm55 | (AC)_11_G(CA)_6_ | AAATTACACCTCTATTTATCCCTG GTGGGAGTGAATGGATTTGTC | 51 | 14/9.1 | 145-201 | 0.90 | 0.91 | 0.88 | 0.503 | JX102626 |
| Spm56 | (CAC)_8_ | GAAGCGAGGGTCGGAGAA TTAGTCTGATTTGAAGGGAAGTG | 53 | 4/1.5 | 153-168 | 0.19 | 0.34 | 0.31 | 0.000^*^ | JX102627 |
| Spm57 | (GT)_12_ | GTTGCATCTCAGGCAGTG TGAAGAGGCTGAAACAAG | 55 | 8/6.1 | 145-165 | 0.59 | 0.85 | 0.82 | 0.000^*^ | JX102628 |
| Spm58 | (TGA)_6_ | TTGACATGGACCCATTCA AGGAGGCTTCTCATCTTT | 53 | 3/2.4 | 281-293 | 0.87 | 0.59 | 0.49 | 0.000^*^ | JX102629 |
| Spm59 | (TGC)_10_ | GTGGGTGATGCTGGATGT GCAAATGGCTGGAATGAA | 53 | 6/5.6 | 271-292 | 0.94 | 0.84 | 0.80 | 0.032 | JX102630 |
| Spm60 | (GT)_7_ | GGCATAGTACAGTTGACGC CATAGCACTTGTCTCCCT | 55 | 6/4.1 | 173-187 | 1.00 | 0.77 | 0.72 | 0.000^*^ | JX102631 |
| Spm61 | (GT)_11_ | TTCACCAAGAAACTAAGCG CACCTCAGATCACAGCGT | 51 | 7/5.4 | 290-308 | 0.77 | 0.83 | 0.79 | 0.467 | JX102632 |
| Spm62 | (ATAC)_11_ | CGGTTATTCCTGTTATGA TGTCTAGCTGTTTCGTAC | 49 | 9/7.5 | 184-228 | 0.94 | 0.88 | 0.85 | 0.175 | JX102633 |
| Spm63 | (TG)_9_ | TTCCATCCCTCATTCCATT TTCCCAGCAGTGCTCACA | 50 | 4/2.4 | 144-158 | 0.28 | 0.59 | 0.50 | 0.001 | JX102634 |
| Mean | / | / | / | 7/4.4 | / | 0.68 | 0.70 | 0.66 | / | / |

*T*_a_, annealing temperature; *N*_a_, observed number of alleles; *N*_e_, effective number of alleles; *H*_O_, observed heterozygosity; *H*_E_, expected heterozygosity; PIC, polymorphism information content; *P*, Chi-square tests for Hardy-Weinberg equilibrium (HWE) after Bonferroni correction (adjusted *P* value = 0.00039); ^*^, significant.
